# Does hemofiltration protect the brain after head trauma? An experimental study in rabbits

**DOI:** 10.1186/s40635-020-00357-5

**Published:** 2020-11-18

**Authors:** Enrique Martinez-Gonzalez, Dolores Garcia-Olmo, Empar Mayordomo-Aranda, Maria Granada-Picazo, Monica Gomez-Juarez, Jeronimo Moreno-Cuesta

**Affiliations:** 1 Experimental Research Unit, Albacete General Hospital, Albacete, Spain; 2 Arnau de Vilanova Hospital, Valencia, Spain; 3 Department of Intensive Care, North Middlesex Hospital, London, UK

**Keywords:** Traumatic brain injury, Continuous veno-venous hemofiltration, Rabbits

## Abstract

**Background:**

Traumatic brain injury (TBI) is one of the most frequent and severe neurological diseases. In the last few decades, significant advances have been made in TBI pathophysiology and monitoring, however new treatments have not emerged. Although the central nervous system (CNS) has been historically defined as an immunologically privileged organ, recent studies show the increasingly predominant role of inflammatory and apoptotic phenomena in the pathogenesis of TBI. Inflammatory response mediators can be eliminated with continuous renal replacement therapies (CRRT). Our aim was to investigate whether hemofiltration protects the brain after head trauma in an experimental study in animals.

**Methods and results:**

A model of TBI and CVVH was performed in anesthetized New Zealand white rabbits without acute renal failure. The experimental group TBI ( +)-CVVH ( +) was compared with a TBI ( +)-CVVH (−) and a TBI (−)-CVVH ( +) control groups. Rabbits were assessed immediately (NES1) and 24 h hours after (NES2) TBI and/or CVVH using a functional Neurological Evaluation Score (NES) and histology of the brains after sacrifice. There was evidence to support a difference of NES1 comparing with the TBI (−)-CVVH ( +), but not with TBI ( +)-CVVH (−) with only 15% of the rabbits treated with CVVH and TBI showing a favorable neurological course. The final neurological outcome (mortality at 24 h) was 0%, 22% and 53% in the TBI(−) + CVVH( +), TBI( +)-CVVH(−) and TBI( +)-CVVH( +) groups respectively. The use of hemofiltration before or after TBI did not make a difference in regards the outcome of the rabbits. There was evidence in the histology to support an increase of mild ischemia, hemorrhage and edema in the experimental group compared with the other two groups.

**Conclusions:**

CVVH in rabbits without renal failure used with the intention to protect the brain may worsen the prognosis in TBI.

## Introduction

Traumatic brain injury (TBI) is one of the most frequent and severe neurological diseases, resulting in high rates of mortality and morbidity [1–3]. This condition not only leaves patients with significant neurological sequelae and disability [1], it also generates an enormous increase in potential years of life lost [4–6], social, economic and emotional impact [7].

Significant advances have been made in TBI pathophysiology in the last few decades as well as in our ability to monitor multiple physiological variables in the laboratory and patient’s bedside. However, there has been little therapeutic advancement. We can attribute the decreased mortality more to adherence to international guidelines for critical care and surgery than to new treatments [4].

The central nervous system (CNS) has been historically defined as an immunologically privileged organ, however recent studies have revealed that it is an important source of inflammatory mediators [8]. Furthermore, studies related to neuro-inflammation following TBI show the increasingly predominant role played by these inflammatory phenomena in the pathogenesis of TBI [9, 10]. Closely related to these neuro-inflammatory events is apoptosis, a phenomenon first studied a few decades ago related to cell death in acute brain damage. Apoptosis complicates brain lesions, regardless of whether they are caused by TBI, brain hemorrhage or stroke [11]. It is possible that in the future, treatments modulating the immune response and apoptosis will be valid therapeutic alternatives for brain damaged patients. Currently, we know that many inflammatory response mediators can be eliminated with continuous renal replacement therapies (CRRT), particularly with convective techniques such as continuous veno-venous hemofiltration (CVVH) arguably indicating a possible immunomodulatory effect upon the inflammatory response [12].

The objective of the current study was to describe the putative protective effect of CVVH in an experimental TBI model in rabbits without acute kidney injury (AKI).

## Materials and methods

### Animals and husbandry

The experimental procedure was in accordance with the Spanish legislation (Royal Decree 1201/2005) approved by the University of Castilla-La Mancha’s Ethical Committee for Animal Experimentation. The manuscript adheres to ARRIVE (Animal Research Reporting of In Vivo Experiments) guidelines.

28 adult females New Zealand White rabbits (Lagomorfa cuniculus) weighing between 3.6 and 6 kg and mean age 6 months (range 4–12 months), were employed. They were housed in the animal facilities at the Albacete General Hospital Research Unit. Environmental conditions and animal handling complied with current regulations (Royal Decree 1201/2005 and Community Guidelines for experimental animals 86/609/CEE). Throughout the experiment, the animals were housed exclusively in individual standard cages, were fed a standard diet for their species (Harlan Interfauna Ibérica; Barcelona) and were given ad libitum access to water. A veterinary doctor gave the rabbits daily examinations until they were euthanized by terminal anesthesia (intracardiac sodium thiopental in previously anesthetized rabbits).

We used rabbits because CVVH devices available were not able to treat smaller animals, and larger animals were more difficult to care and handle at our Research Unit.

### Anesthetic protocol

The anesthetic protocol [13, 14] was initiated with an intramuscular administration of ketamine (dose of 35 mg/kg) and xylazine (7 mg/kg) in the animal’s hind legs, aiming to produce anesthesia without loss of spontaneous breathing. Once the righting reflex was lost, the ears, tail, head and both groin areas were shaved. Next, in order to mechanically ventilate the rabbit and maintain anesthesia with inhalation agents, we proceeded with orotracheal intubation (OTI); an endotracheal pediatric tube was employed with an internal diameter of 3.5 mm, previously lubricated and without a cuff. We used the inhalation agent isoflurane, without nitrous oxide; the fraction of inspired oxygen was 100%. In order to maintain the anesthetic coma while keeping hemodynamic parameters within normal limits [15, 16] isoflurane doses were adjusted according to the animal´s clinical response and to the values observed with hemodynamic monitoring. The isoflurane concentrations administered oscillated between 1 and 2%. During mechanical ventilation (MV) (Datex-Engström EAS 9010, GE Healthcare, USA) and subsequent procedures, the following respiratory parameters were monitored [15, 16] to ensure normal respiratory values: oxygen saturation (O_2_Sat) using a pulse-oximeter (Ohmeda 3740, Datex-Ohmeda, GE Healthcare, Illinois, USA) placed on the shaved tail of the animal; carbon dioxide at the end of exhalation (EtCO_2_), tidal volume (*V*_T_) and respiratory rate (RR) using a respiratory monitor (Datex Capnomac Ultima, Datex-Engstrom, Helsinki, Finland). Next, in order to administer maintenance fluid therapy consisting of normal saline solution (NSS, Fresenius Kabi, Barcelona, Spain), we proceeded to cannulate the marginal ear vein using 24G intravenous catheters (Abbocath, Hospira, Donegal Town, Ireland). In the same way, we cannulated the central ear artery for invasive hemodynamic monitoring of blood pressure (BP) and heart rate (HR) throughout the entire procedure. We considered normal hemodynamic and respiratory values [15, 16] to be the following: BP 100/70 (85) mmHg, HR 180–300 bpm, O_2_Sat > 90%, and EtCO2 30–35 mmHg.

### Study groups

**Fig. 1:**
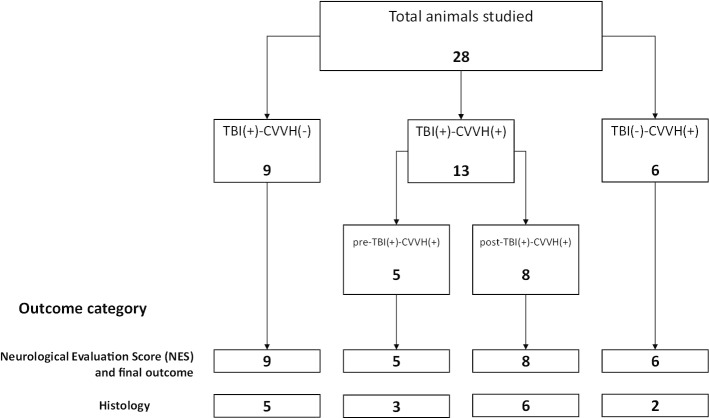
Experimental design

A consecutive allocation of animals were clustered within three groups as shown in Fig. 1. Traumatic brain injury or TBI ( +)-CVVH (−) group (*n* = 9) which included rabbits with a positive TBI (based on the presence of neurological and hemodynamic signs described later) that received no CVVH treatment. Continuous veno-venous hemofiltration or TBI (−)-CVVH ( +) group (*n* = 6) consisting of the rabbits that received CVVH therapy without being subjected to TBI. Finally a combined TBI and CVVH experimental or TBI ( +)-CVVH( +) group (*n* = 13) which included all of the animals subjected to TBI and that also received CVVH therapy. This last group included two subgroups, namely rabbits with hemofiltration immediately before the TBI or pre-TBI ( +)-CVVH ( +) (*n* = 5) and rabbits with hemofiltration immediate after the TBI or post-TBI ( +)-CVVH( +) (*n* = 8).

### Traumatic brain injury model

Our model of TBI was an original modification of the lateral fluid-percussion model by McIntosh et al. [17] and is showed in Additional file 1: Figure S1. Once anesthetized, monitored and connected to MV, we placed the rabbit in prone position and prepared a sterile field in the shaved area of the head. A midline incision was made in the skin, and the tissues of the head were dissected until we reached bone tissue. Next, with a digital caliper (Centigraff, Barcelona, Spain) we located a point on the right hemisphere of the skull, situated 16.5 mm caudal to the anterior suture, and 2.4 mm lateral to the sagittal suture. Anatomically, this point was at the level of the outer canthus of the rabbit´s eye and slightly lateral to the sagittal suture. We then proceeded with trepanation, using a hand drill with a 2.5 mm bore; the walls of the resulting orifice were coated with bone wax (Ethicon Inc, USA). An intravenous (IV) 16G catheter (Introcan, Braun, Melsungen, Germany), whose length was modified such that the cone remained in contact with the cranium, and the distal end of the cannula with the dura mater, was placed in the orifice. Small incisions were made in the periosteum of the cranium around the orifice, after which the area was thoroughly dried and the catheter fixed to the animal´s skull using a sterile adhesive (Dermabond, Ethicon Inc, USA). In this way, the skull remained leak-proof, preventing fluid from escaping through the trepanation orifice at the moment of impact; this insured sufficient intracranial pressure to cause TBIs of similar quality in all animals.

In order to produce the impact, we used an intravenous contrast injector (Injektron 82 M, Medtron AG, Saarbrüken, Germany), which allowed us to inject a known volume of fluid at a constant and controlled rate. Through an IV infusion system (Perfusend, Sendal, Caceres, Spain), the contrast injector syringe was connected to the IV catheter inserted into the trepanation orifice. Before the impact, we administered 5 μg/kg of fentanyl in order to provide analgesia and avoid sudden movements that could result in accidental extubation or loss of the IV cannulas upon impact. Normo-saline solution (NSS) was employed to produce the TBI. In a series of preliminary experiments, we determined the ideal injectable volume of NSS necessary to produce a clinically significant brain lesion without immediately compromising the life of the animal. This was done by administering different volumes (10, 7, 5 and 3 ml) to different animals, at a velocity of 7.5 ml/s (the maximum speed allowed by our injector), and observing clinical response and course. We finally concluded that 5 ml of NSS was the ideal volume with which to conduct our study.

### Continuous veno-venous hemofiltration model

To prepare for CVVH treatment, the CVVH machine (Aquarius, Nikkiso, Langenhagen, Germany) was set up by positioning the bags of replacement solution (Accusol 35, Baxter, IL, USA), the collection bags, the CVVH tubing and the hemofilter (polyethersulfone membrane filter 0.3 m^2^, HF03, Baxter, IL, USA), after which the entire system was purged. Once the machine was ready, the rabbit was placed in supine position, and a sterile field was prepared in the shaved inguinal area; it was generally on the right side for the convenience of the researcher. The femoral artery pulse was palpated, and with a scalpel blade, we made an incision in the skin following the direction of this pulse. Next, planes were dissected until transparency allowed for a clear visualization of the femoral vein, which was cannulated with a 20G intravenous catheter (Abbocath, Hospira, Donegal Town, Ireland). A Seldinger [18] technique was employed, first inserting a guidewire into the vein in order to place a 5 French catheter 6 cm length for hemofiltration (DL5/6, Baxter, IL, USA). Finally, the catheter was sutured (Silkam, Braun, Aesculap AG, Tuttlingen, Germany) to the muscular plane in order to avoid accidental dislodging of it during the procedure, and the surgical wound was left open until the end of the session. Once the catheter was inserted, it was connected to the CVVH system and the treatment was initiated according to the protocol described below. Drops in temperature was prevented using the active blood heater of the Aquarius machine during all experiments. The TBI(−)-CVVH( +) group underwent craniectomy without the fluid pulse. Brains were also histopathological image analyzed.

### Combined TBI and CVVH experimental model

The femoral vein was cannulated in preparation for CVVH, and the TBI-inducing device was set up. Then, two subgroups were established. One subgroup had the hemofiltration for 90 min before the TBI (*n* = 5). In the other subgroup the rabbits were placed prone, and TBI was produced according to the protocol described above, immediately after the procedure, the rabbit was placed supine, catheter was connected to the CVVH system, and treatment was initiated (*n* = 8). The treatment procedure was as follows: CVVH dose 45 ml/kg/h, net fluid loss was individually adjusted to obtain neutral fluid balances in order to avoid brain edema and the effect of fluid therapy on TBI. CVVH was administered without heparin [7, 19] therefore allowing initiation with double connection in order to minimize the possibility of hypotension in the rabbit. The duration of the CVVH session was established in 90 min. In preliminary studies, we observed that in the majority of cases, this was the coagulation time limit beyond which there was a risk of jeopardizing blood return to the rabbit at the end of treatment, with subsequent death. The replacement solution infusion took place in the pre-filter modality, thus diminishing the risk of coagulation and prolonging the effective treatment time. Noradrenaline (Braun, Tuttlingen, Germany) was administered as needed (0–1.6 μg/kg/min) in order to reach the target mean arterial blood pressure (MBP) of at least 45 mmHg.

### Neurological assessment model

We developed a novel Neurological Evaluation Score (NES) with 5 parameters to clinically evaluate the animals. The score measured the ability to jump and/or spatial orientation in the air, the ability to wander freely, the toe spread reflex that is produced when the rabbit is abruptly lifted off the ground, the postural reflex (resistance to lateral pushes) and finally the ability to maintain balance on a 15° inclined plane without falling. Each one of these items was scored between 0 and 2 points, such that the maximum score was 10 points. We considered severe TBI with 0 to 3 points, moderate TBI 4 to 7 points and mild TBI 8 to 10 points. The neurological assessments were performed at three distinct points in time: before TBI (basal NES), immediately following TBI (NES1) and at 24 h (NES2) post-TBI. We established the 24 h observation period based on our preliminary experiments. In these studies, we noted that the first rabbits to undergo TBI had practically regained their basal level neurological status after 48–72 h, in spite of severe TBI scores according to our scale. Therefore, neurological symptomatology was highest at 24 h following brain injury. Additional collected data were clinical (withdrawal movements to pain at the moment of impact; motor disturbance in the form of paraparesis, or hemiparesis, difficulty holding the head upright, deviation of the eyes and head towards the same side of the injury), water consumption at 24 h and weight loss 24 h post-TBI, suggesting minimal or no food consumption.

A positive response to the induced TBI was indicated by the presence of at least two of the following signs in our animals, namely a change in BP and HR of at least 10% from pre-TBI basal values, neurological symptoms immediately following TBI and at 24 h post-injury manifested as motor disturbances of the legs and head, changes in scores on the NES with respect to baseline immediately following the injury and at 24 h, lower than normal levels of water consumption for a rabbit, and weight loss at 24 h post-TBI. The response to TBI was considered negative if the rabbit did not present at least two of the above-named conditions.

The final neurological outcome of the rabbits was considered at 24 h with rabbits either alive (favorable outcome) or dead (unfavorable outcome).

### Histopathology study

24-h post-TBI and following neurological evaluation, the rabbits were anesthetized intramuscularly with 50 mg/kg of ketamine (Ketolar, Pfizer, Barcelona, Spain) and 10 mg/kg of xylazine (Xilagesic 2%, Calier, Barcelona, Spain) such that spontaneous breathing was maintained. Next, the animals were sacrificed by means of an intracardiac injection with sodium thiopental (Braun, Barcelona, Spain) 500 mg diluted with 20 ml of NSS to avoid the pain of injection. Immediately, a craniectomy was performed and the cranial bones removed with the aid of a gouge clamp (REDA, Tuttlingen, Germany). In order for the brain to remain intact after exposure, it was extracted very carefully with surgical instruments and then preserved in a jar of formalin 4% (VWR Eurolab, PA, USA).

After extraction, the brains were examined histopathologically with the collaboration of the Pathology Department of Albacete General Hospital. Initially, we performed macroscopic studies to describe the observed lesions, followed by microscopic examination of finely sliced preparations stained with hematoxylin–eosin. At the macroscopic level, we compared the following items between animals: the presence or absence of injury in the area of impact and the characteristics of the lesion; the presence of bleeding, and brain consistency as an indirect reflection of cerebral edema. Likewise, at the microscopic level, we observed the following characteristics: presence or absence of bleeding and type of hemorrhage [17], characteristics of the area of injury, local and/or distant inflammatory response, and presence or absence of edema, ischemia, gliosis and their characteristics (Additional file 2: Figure S2).

### Statistical analysis

Descriptive analyses were undertaken to characterize the study sample using mean and standard deviation. Statistical modeling took into consideration the distribution of the variables (normality), type of response variable, and repeated measures.

Mann–Whitney test was used for two-group comparisons. Kruskal–Wallis test was performed for more than two group analysis with Dunn test and Bonferroni correction to identify intergroup differences. Contingency tables with Chi-square test and standardized residuals was used for categorical variables.

The R package (R Foundation for Statistical Computing, 2013) was used for statistical analysis [20].

## Results

### Basal data

The basal data for all groups are shown in Table 1. There was no evidence to support a difference between groups, except for the mean heart rate between TBI( +)-CVVH(−) and TBI(−)-CVVH( +).

**Table 1:** Basal data for all groups

Group	Weight	Mean BP	Mean HR	Mean SpO_2_	Mean ETCO_2_
TBI( +)-CVVH(−)	4441.1 (314.3)	62.8 (10.6)	165.7 (24.0)	98.7 (1.3)	31.8 (7.1)
TBI(−)-CVVH( +)	5006.6 (330.0)	61.1 (12.0)	132.3 (29.2)*	99.3 (0.5)	31.3 (8.0)
TBI( +)-CVVH( +)	4751.5 (709.8)	63.2 (23.5)	156.0 (26.0)	98.8 (1.6)	31.0 (5.4)
KW (*p*)	5.4 (0.06)	0.13 (0.93)	6.3 (0.04)	0.15 (0.92)	0.04 (0.97)

Values expressed as $\overline{{\varvec{x}}}$±SD

*TBI(+)-CVVH(−)* traumatic brain injury group (*n* = 9), *TBI(−)-CVVH( +)* hemofiltration group (*n* = 6). *TBI( +)-CVVH( +)* combined traumatic brain injury group adding all rabbits with hemofiltration before and after the trauma (*n* = 13), *KW* Kruskal–Wallis test

*p = 0.017 between TBI( +)-CVVH(−) and TBI(−)-CVVH( +) using Dunn test

### Physiological data

The physiological variables are shown in Table 2 and their comparisons in Additional file 3: Table S1. There was no evidence to support a difference between groups with regard to O_2_Sat and EtCO_2._ The TBI( +)-CVVH( +) group had lower BP at 15 and 30 min post-intervention, and higher heart rate at 60 min post-intervention.

**Table 2:** Physiological variables by groups

	TBI( +)-CVVH(−) (*n* = 9)				TBI(−)-CVVH( +) (*n* = 6)				PreTBI( +)-CVVH( +) (*n* = 5)				PostTBI( +)-CVVH( +) (*n* = 8)				TBI( +)-CVVH( +) (*n* = 5 + 8 = 13)			
Times	MBP	HR	SO_2_	ETCO_2_	MBP	HR	SO_2_	ETCO_2_	MBP	HR	SO_2_	ETCO_2_	MBP	HR	SO_2_	ETCO_2_	MBP	HR	SO_2_	ETCO_2_
T1	62.8 (10.6)	165.7 (24)	98.7 (1.3)	31.8 (7.1)	61.1 (12)	132.3 (29.2)	99.3 (0.5)	31.3 (8)	68.8 (10.4)	166.8 (24.8)	98 (2.4)	33.2 (5.8)	59.7 (29.1)	149.2 (26)	99.3 (0.5)	29.5 (5)	63.2 (23.5)	156 (26)	98.8 (1.6)	31 (5.4)
T2	87 (18.7)	176.8 (49.9)	96.7 (6.3)	26.1 (3.2)	34.8 (10.5)	118.8 (16.1)	98.3 (2)	30 (11.7)	37 (16.3)	152 (26.8)	99.5 (0.7)	29.2 (2.7)	48.6 (24.7)	136.5 (25.9)	97.2 (5.1)	28 (11.1)	44.1 (21.9)	142.4 (26.3)	97.8 (4.3)	28.5 (8.4)
T3	81.1 (10.7)	176.5 (40.9)	97.1 (4.6)	28.2 (4.4)	34 (11.2)	106 (17.5)	98.5 (2.1)	29.5 (12)	36.8 (11.6)	145.4 (19.3)	98.5 (0.7)	37 (7.2)	39.7 (17.6)	128.7 (24.6)	98.6 (1.6)	24.1 (8.9)	38.6 (15.1)	135.1 (23.4)	98.5 (1.3)	28.4 (10.1)
T4	(.)	(.)	(.)	(.)	32.4 (11.9)	108.8 (18.4)	60 (.)	32 (.)	41.4 (11.8)	145.2 (18.5)	99 (0)	35 (11.3)	44 (22.3)	127 (28.3)	99 (1)	25.5 (8.5)	42.8 (17.5)	135.2 (25.1)	99 (0.7)	28.6 (9.6)
T5	(.)	(.)	(.)	(.)	27.6 (10)	110.3 (5.8)	99 (.)	33 (.)	43.2 (11.9)	141.2 (20.9)	99 (.)	44 (.)	40.6 (17.4)	127.8 (19.3)	98.5 (2.1)	29.3 (3.5)	41.9 (14.1)	134.5 (20.2)	98.6 (1.5)	33 (7.8)

Values expressed as ($\overline{{\varvec{x}}}$±SD). T1 = basal, T2 = at 15 min, T3 = at 30 min, T4 = at 60 min and T5 = at 90 min

*TBI( +)-CVVH(−)* traumatic brain injury group, *TBI(−)-CVVH( +)* hemofiltration group, *PreTBI( +)-CVVH( +)* traumatic brain injury group with hemo-filtration before the trauma, *PostTBI( +)-CVVH( +)* traumatic brain injury with hemofiltration post-trauma, *TBI( +)-CVVH( +)* combined group adding PreTBI( +)-CVVH( +) and PostTBI( +)-CVVH( +), *MBP* mean arterial blood pressure (mm Hg), *HR* heart rate (b/min). *SO*_*2*_ peripheral arterial oxygen saturation (%), *ETCO*_*2*_ end-tidal carbon dioxide (mm Hg)

### Hemofiltration time

We did not find differences between the control hemofiltration group, TBI(−) + CVVH( +), and the experimental group, TBI( +)-CVVH( +), with regard to total time of hemofiltration (74.1 ± 29.5 versus 105 ± 53, *p* = 0.14) and efficient time of hemofiltration (57.5 ± 36.1 versus 74.2 ± 27.9, *p* = 0.36) (Additional file 3: Table S2).

### Neurological evaluation

*The NES immediately (NES1) and 24 h* (NES2) is shown in Additional file 3: Table S3. There was evidence to support a difference of NES1 comparing with TBI(−)-CVVH( +), but not with TBI( +)-CVVH(−). The lack of difference in the neurological evaluation between TBI( +)-CVVH( +) and TBI( +)-CVVH(−) continue to NES2.

The final neurological outcome (mortality at 24 h) was 0%, 22% and 53% in the TBI(−) + CVVH( +), TBI( +)-CVVH(−) and TBI( +)-CVVH( +) groups, respectively. There is evidence of a more frequent unfavorable outcome in the TBI( +)-CVVH( +) group as shown in Table 3. The use of hemofiltration before or after TBI did not make a difference with regard to the outcome of the rabbits (*χ*^2^ = 0, *df* = 1, *p* = 1).

**Table 3:** Proportional table (%) between groups by final neurological outcome

Outcome	TBI( +)-CVVH(−)	TBI(−)-CVVH( +)	TBI( +)-CVVH( +)
Unfavorable	21.4	0.0	78.5
Favorable	42.8	42.8	14.2*

*TBI( +)-CVVH(−)* traumatic brain injury group (*n* = 9), *TBI(-)-CVVH( +)* hemofiltration group (*n* = 6), *TBI( +)-CVVH( +)* combined traumatic brain injury group adding all rabbits with hemofiltration before and after the trauma (*n* = 13). Pearson’s chi-squared. *Denotes a significant standardized residual

### Histopathology

**Fig. 2:**
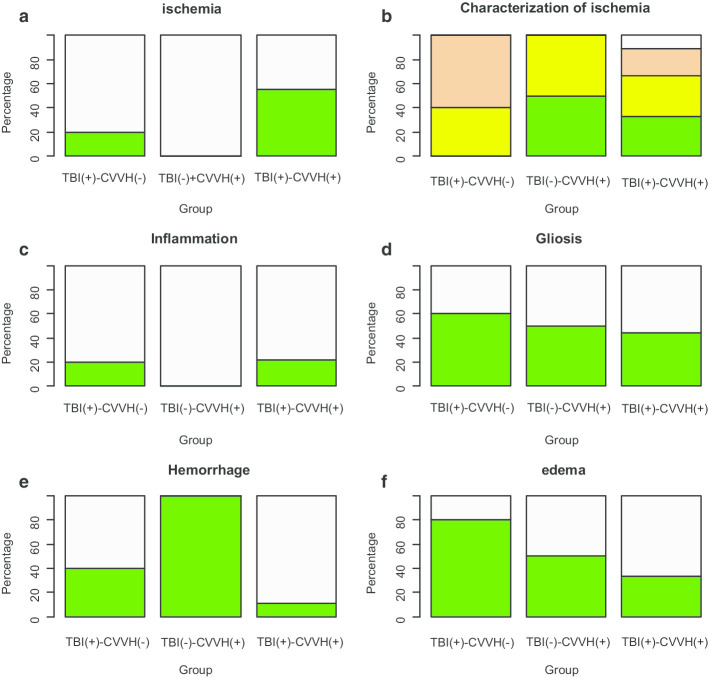
Pathology within groups. **a** Areas of ischemia, green = mild ischemia, grey = severe ischemia. **b** Location of ischemia, green = isolated and diffuse, grey = multiple and focal, brown = multiple and diffuse, yellow = isolated and focal. **c** Green = normal areas without inflammation, grey = presence of inflammation. **d** Green = normal areas without gliosis, grey = presence of gliosis. **e** Green = areas without hemorrhage, grey = presence of hemorrhage. **f** Green = areas without edema, grey = presence of edema. TBI( +)-CVVH(−) = traumatic brain injury group (*n* = 5). TBI(−)-CVVH( +) = hemofiltration group (*n* = 2). TBI( +)-CVVH( +) = combined traumatic brain injury group adding all rabbits with hemofiltration before and after the trauma (*n* = 9)

The histopathology finding within groups is shown in Fig. 2 and comparisons between groups in Additional file 3: Table S4. There was evidence in the histology findings to support an increase in the TBI( +)-CVVH( +) group of mild ischemia, hemorrhage and edema compared with the other groups.

## Discussion

We have demonstrated that the use of hemofiltration to protect a traumatic brain in rabbits without renal failure induces more unfavorable outcome than the TBI per se. Only 15% of the rabbits allocated to the model of TBI and hemofiltration presented a favorable clinical course, were extubated and experienced a mild improvement in neurological condition at 24 h following injury in contradistinction with an 85% that showed an unfavorable clinical course. Furthermore, a significantly greater number of experimental group animals experienced an unfavorable clinical course compared with animals of control groups. The histopathological analysis demonstrated that in our experimental model more rabbits developed mild ischemia, hemorrhage and edema compared with the other groups. These data support that hemofiltration has no beneficial effect on mortality or neurological prognosis in an experimental TBI model in rabbits without AKI.

Nonetheless, various factors may have influenced our results. Firstly, it is possible that the hemofiltration dose employed in the current study were insufficient to produce the desired effect, although in humans there is no evidence to date that doses higher than 20–25 ml/kg/h in sepsis (an inflammatory response syndrome) increase survival in humans [21–23]. Secondly, in our study, the administration of CVVH without anticoagulants shortened treatment time, such that it may have been insufficient to achieve the desired outcome. Thirdly, also due to the absence of anticoagulants, there were frequent interruptions during treatment sessions, which caused discrepancies between the prescribed doses and the actual doses administered to the rabbits. Finally, the low BP in the experimental group (TBI( +)-CVVH( +)) may have been another factor which negatively affected our results. Low BP interferes with proper functioning of CRRT, causing frequent interruptions to therapy, which reduce effective treatment time and lower the actual dose administered. Furthermore, low BP worsens the prognosis of TBI patients by diminishing cerebral perfusion pressure and cerebral blood flow. These data may corroborate the deleterious effects of low BP on TBI, and at the same time, reveal that low BP could be a confounding factor in this study. Furthermore, the use of the hemofiltration before or after the TBI did not improve the neurological outcome. The existence of uncouple proteins that decreases the mitochondrial production of reactive oxygen species during acute stress responses (e.g., myocardial and brain ischemia) was the fundamental basis for using the hemofiltration as an stressor before the TBI, trusting a stress preconditioning over the body and brain may happen [24]. Unfortunately, there was no evidence of a potential preconditioning effect of hemofiltration over the brain as demonstrated for equal outcomes in both experimental subgroups (hemofiltration pre-TBI and hemofiltration post-TBI).

The study has some limitations. Firstly, the study is descriptive and pragmatic in its conception. The efficacy of the renal replacement therapy was neither researched with biomarkers, nor inflammatory mediators, blood electrolyte concentration or cerebral perfusion pressure. Secondly, the election of clinical evaluation at 24 h (NES at 24 h) could suggest that the score was not able to identify a severe TBI because some of the rabbits were nearly back to normal in 48–72 h. Nevertheless, evaluation within the first 24 h of admission is a current paradigm in predictive models for the critically ill patient, such as ICNARC or APACHE scores. Furthermore, neurological evaluation, for example with GCS, is never completely accurate as demonstrated with the need of additional factors to match observed mortality in humans [25].

We cannot conclude that CVVH has a beneficial effect on prognosis for TBI. Another question is whether our observations provide evidence of the opposite, namely that CVVH may have negative effects on TBI. Of the different schools of thought that attempt to explain the stress response in critically ill patients, the post-modernist school [24] establishes a balance between the traditionalist and modernist schools, adopting the essential concept of allostasis, meaning stabilization of the organism through change. This model explains that the brain anticipates the most likely demand during a stress response and modifies its physiological variables in order to meet the anticipated demand. Therefore, a pharmacological or mechanical intervention such as hemofiltration, employed at an inappropriate time during the stress response, may alter the brain´s anticipatory response and prove to be inadequate or even deleterious. Furthermore, these interventions can be considered stressors as such, which may aggravate the stress of the existing acute insult and have negative consequences for the patient. However, since our study was not designed to determine whether CVVH has negative effects on TBI, and due to limiting factors such as low BP, we cannot draw general conclusions with respect to this issue.

## Conclusion

Continuous veno-venous hemofiltration in rabbits without renal failure used with the intention to protect the brain may worsen the prognosis in traumatic brain injury.

## Supplementary information

**Additional file 1: Figure S1.** Lateral fluid-percussion TBI model.

**Additional file 2: Figure S2.** Histopathological lesions (A to E) *from TBI(* +*)-CVVH(* +*)*: A: Gliosis and inflammation. B: Gliosis and edema. C: Gliosis and edema. D: Hemorrhages. E: Ischemia and inflammation. *F*: Exemplar of a whole brain section in a rabbit with TBI( +)-CVVH(-)

**Additional file 3: Table S1.** Mean BP (mmHg) by groups. BP1 = basal,BP2 = at 15 min, BP3 = at 30 min, BP4 = at 60 min and BP5 = at 90 min. *p = 0.0018 between TBI( +)-CVVH(-) and TBI(-)-CVVH( +). †*p* = 0.0022 between TBI( +)-CVVH(-) and TBI( +)-CVVH( +) using KW with Dunn test. Mean HR (b/min) by groups. HR1 = basal, HR2 = at 15 min, HR3 = at 30 min, HR4 = at 60 min and HR5 = at 90 min. *p = 0.017 (HR1), 0.007 (HR2), 0.0004 (HR3) between TBI( +)-CVVH(-) and TBI(-)-CVVH( +); †*p* = 0.007 (HR4), 0.01 (HR5) between TBI(-)-CVVH( +) and TBI( +)-CVVH( +) using Dunn test. Mean SpO2(%) by groups. SpO2-1 = basal, SpO2-2 = at 15 min, SpO2-3 = at 30 min, SpO2-4 = at 60 min and SpO2-5 = at 90 min. Mean ETCO2 (mmHg) by groups. ETCO2-1 = basal, ETCO2-2 = at 15 min, ETCO2-3 = at 30 min, ETCO2-4 = at 60 min and ETCO2-5 = at 90 min. Values expressed as $\overline{{x}}$ (SD). TBI( +)-CVVH(-) = Traumatic brain injury group (*n* = 9). TBI(-)-CVVH( +) = Hemofiltration group (*n* = 6). TBI( +)-CVVH( +) = Combined traumatic brain injury group adding all animals with hemofiltration before and after the trauma (*n* = 13). KW = Kruskal–Wallis test. NA = Not available. **Table S2.** Hemofiltration time. TT = Total time of hemofiltration (min). ET = efficient time of hemofiltration (min). TBI(-)-CVVH( +) = Hemofiltration control group. TBI( +)-CVVH( +) = Traumatic brain injury group with hemofiltration. Values expressed as $\overline{{{x}}}$(SD). MW = Mann–Whitney test. **Table S3.** Neurological Evaluation Score (NES) immediately (NES1) and 24 h (NES2) after TBI for all groups. Values expressed as $\overline{{x}}$±SD. TBI( +)-CVVH(-) = Traumatic brain injury group (*n* = 9). TBI(-)-CVVH( +) = Hemofiltration group (*n* = 6). TBI( +)-CVVH( +) = Combined traumatic brain injury group adding all rabbits with hemo-filtration before and after the trauma (*n* total = 13). *p = 0.0020 between TBI( +)-CVVH(-) and CV (*n* = 6) †*p* = 0.02 between TBI(-)-CVVH( +)and TBI( +)-CVVH( +) using KW with Dunn test. KW = Kruskal–Wallis test. **Table S4.** Proportional table (%) showing Pearson’s chi-squared between groups for the presence of cerebral ischemia (*p* < 0.001), inflammation (*p* < 0.001), gliosis (*p* = 0.14), hemorrhage (*p* < 0.001) and edema (*p* < 0.001). *Denotes a significant standardized residual. TBI( +)-CVVH(-) = Traumatic brain injury group (*n* = 5). TBI(-)-CVVH( +) = Hemofiltration group (*n* = 2). TBI( +)-CVVH( +) = Combined traumatic brain injury group adding all rabbits with hemo-filtration before and after the trauma (*n* = 9).

## Data Availability

The data sets used and/or analyzed during the current study are available from the corresponding author upon reasonable request. Likewise, all of the data generated or analyzed in this study are included in this published article (and its Additional files).

## Abbreviations

AKI: Acute kidney injury
BP: Blood pressure
CNS: Central nervous system
CRRT: Continuous renal replacement therapies
TBI ( +)-CVVH (−): Control traumatic brain injury group
TBI (−)-CVVH ( +): Control veno-venous hemofiltration group
TBI ( +)-CVVH ( +): Experimental group including animals with the CVVH before and after TBI
Pre-TBI ( +)-CVVH ( +): Experimental group with CVVH before the TBI
Post-TBI ( +)-CVVH ( +): Experimental group with CVVH after TBI
CVVH: Continuous veno-venous hemofiltration
EtCO_2_: Carbon dioxide at the end of exhalation
HR: Heart rate
IV: Intravenous
MBP: Mean blood pressure
MV: Mechanical ventilation
NES: Neurological evaluation score
NSS: Normal saline solution
O_2_Sat: Oxygen saturation
OTI: Orotracheal intubation
PIP: Peak inspiratory pressure
RR: Respiratory rate
TBI: Traumatic brain injury
*V*_T_: Tidal volume
