# Supplementary figures and images for: Does hemofiltration protect the brain after head trauma? An experimental study in rabbits
Source: Intensive Care Med Exp. 2020 Nov 18;8:66. doi: 10.1186/s40635-020-00357-5 (PMC7674531; doi:10.1186/s40635-020-00357-5)

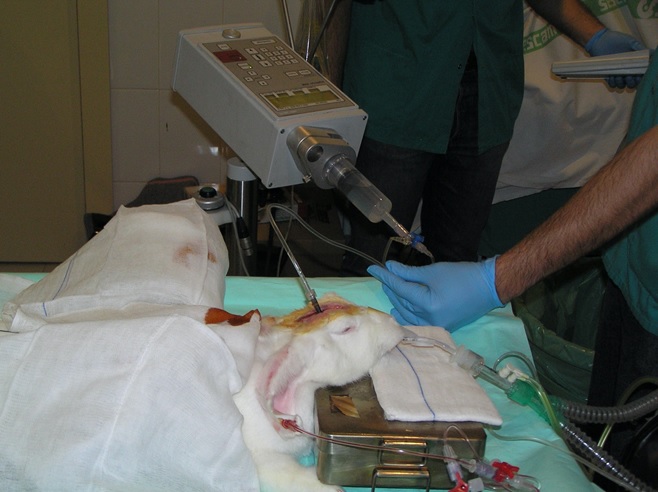

Supplement: Supplementary file 1 — Additional file 1: Figure S1. Lateral fluid-percussion TBI model. [file 40635_2020_357_MOESM1_ESM.jpg]

## Slide 1
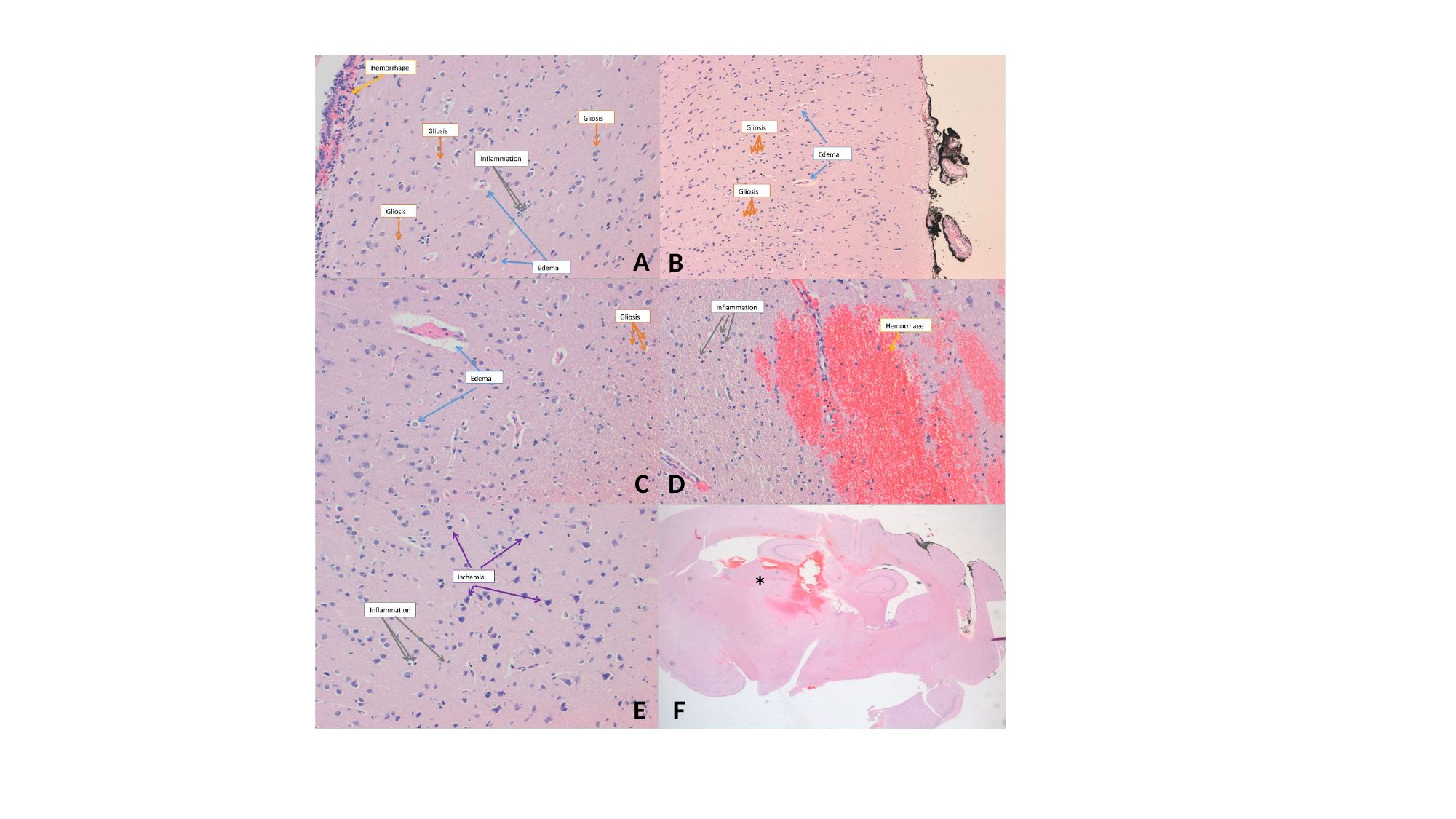

A
B
C
D
*
E
F
F

Supplement: Supplementary file 2 — Additional file 2: Figure S2. Histopathological lesions (A to E) from TBI( +)-CVVH( +): A: Gliosis and inflammation. B: Gliosis and edema. C: Gliosis and edema. D: Hemorrhages. E: Ischemia and inflammation. F: Exemplar of a whole brain section in a rabbit with TBI( +)-CVVH(-) [file 40635_2020_357_MOESM2_ESM.pptx]
