# Supplementary material for: Does hemofiltration protect the brain after head trauma? An experimental study in rabbits
Source: Intensive Care Med Exp. 2020 Nov 18;8:66. doi: 10.1186/s40635-020-00357-5 (PMC7674531; doi:10.1186/s40635-020-00357-5)
Supplement: Supplementary file 3 — Additional file 3: Table S1. Mean BP (mmHg) by groups. BP1 = basal,BP2 = at 15 min, BP3 = at 30 min, BP4 = at 60 min and BP5 = at 90 min. *p = 0.0018 between TBI( +)-CVVH(-) and TBI(-)-CVVH( +). †p = 0.0022 between TBI( +)-CVVH(-) and TBI( +)-CVVH( +) using KW with Dunn test. Mean HR (b/min) by groups. HR1 = basal, HR2 = at 15 min, HR3 = at 30 min, HR4 = at 60 min and HR5 = at 90 min. *p = 0.017 (HR1), 0.007 (HR2), 0.0004 (HR3) between TBI( +)-CVVH(-) and TBI(-)-CVVH( +); †p = 0.007 (HR4), 0.01 (HR5) between TBI(-)-CVVH( +) and TBI( +)-CVVH( +) using Dunn test. Mean SpO2(%) by groups. SpO2-1 = basal, SpO2-2 = at 15 min, SpO2-3 = at 30 min, SpO2-4 = at 60 min and SpO2-5 = at 90 min. Mean ETCO2 (mmHg) by groups. ETCO2-1 = basal, ETCO2-2 = at 15 min, ETCO2-3 = at 30 min, ETCO2-4 = at 60 min and ETCO2-5 = at 90 min. Values expressed as $\overline{{x}}$x¯ (SD). TBI( +)-CVVH(-) = Traumatic brain injury group (n = 9). TBI(-)-CVVH( +) = Hemofiltration group (n = 6). TBI( +)-CVVH( +) = Combined traumatic brain injury group adding all animals with hemofiltration before and after the trauma (n = 13). KW = Kruskal–Wallis test. NA = Not available. Table S2. Hemofiltration time. TT = Total time of hemofiltration (min). ET = efficient time of hemofiltration (min). TBI(-)-CVVH( +) = Hemofiltration control group. TBI( +)-CVVH( +) = Traumatic brain injury group with hemofiltration. Values expressed as $\overline{{{x}}}$x¯(SD). MW = Mann–Whitney test. Table S3. Neurological Evaluation Score (NES) immediate [file 40635_2020_357_MOESM3_ESM.docx]

**Supplemental Table 1.** Mean BP (mmHg) by groups. BP1=basal,BP2=at 15 min, BP3=at 30 min, BP4=at 60 min and BP5=at 90 min. *p = 0.0018 between TBI(+)-CVVH(-) and TBI(-)-CVVH(+). †p = 0.0022 between TBI(+)-CVVH(-) and TBI(+)-CVVH(+) using KW with Dunn test. Mean HR (b/min) by groups. HR1=basal, HR2=at 15 min, HR3=at 30 min, HR4=at 60 min and HR5=at 90 min. *p = 0.017 (HR1), 0.007 (HR2), 0.0004 (HR3) between TBI(+)-CVVH(-) and TBI(-)-CVVH(+); †p=0.007 (HR4), 0.01 (HR5) between TBI(-)-CVVH(+) and TBI(+)-CVVH(+) using Dunn test. Mean SpO_2_(%) by groups. SpO_2_-1=basal, SpO_2_-2=at 15 min, SpO_2_-3=at 30 min, SpO_2_-4=at 60 min and SpO_2_-5=at 90 min. Mean ETCO_2_(mmHg) by groups. ETCO_2_-1=basal, ETCO_2_-2=at 15 min, ETCO_2_-3=at 30 min, ETCO_2_-4=at 60 min and ETCO_2_-5=at 90 min. Values expressed as $\overline{\boldsymbol{x}}$(SD). TBI(+)-CVVH(-) = Traumatic brain injury group (n=9). TBI(-)-CVVH(+) = Hemofiltration group (n=6). TBI(+)-CVVH(+)= Combined traumatic brain injury group adding all animals with hemofiltration before and after the trauma (n =13). KW= Kruskal-Wallis test. NA = Not available

| GROUP | BP1 | BP2 | BP3 | BP4 | BP5 |
| --- | --- | --- | --- | --- | --- |
| TBI(+)-CVVH(-) | 62.8 (10.6) | 87.0 (18.7) | 81.1 (10.7) | NA | NA |
| TBI(-)-CVVH(+) | 61.1 (12.0) | 34.8 (10.5)* | 34.0 (11.2)* | 32.4 (11.9) | 27.6 (10.0) |
| TBI(+)-CVVH(+) | 63.2 (23.5) | 44.1 (21.9)† | 38.6 (15.1)† | 42.8 (17.5) | 41.9 (14.1) |
| KW (p) | 0.13 (0.93) | 13.8 (0.0009) | 16.9 (0.0002) | 1.1 (0.2) | 2.3 (0.13) |
|  | **HR1** | **HR2** | **HR3** | **HR4** | **HR5** |
| TBI(+)-CVVH(-) | 165.7 (24.0) | 176.8 (49.9) | 176.5 (40.9) | NA | NA |
| TBI(-)-CVVH(+) | 132.3 (29.2)* | 118.8 (16.1)* | 106.0 (17.5)* | 108.8 (18.4) | 110.3 (5.8) |
| TBI(+)-CVVH(+) | 156.0 (26.0) | 142.4 (26.3) | 135.1 (23.4) | 135.2 (25.1)† | 134.5 (20.2)† |
| KW (p) | 6.3 (0.04) | 7.9 (0.02) | 13.3 (0.001) | 5.9 (0.01) | 5.2 (0.02) |
|  | **SpO_2_-1** | **SpO_2_-2** | **SpO_2_-3** | **SpO_2_-4** | **SpO_2_-5** |
| TBI(+)-CVVH(-) | 98.7 (1.3) | 96.7 (6.3) | 97.1 (4.6) | NA | NA |
| TBI(-)-CVVH(+) | 99.3 (0.5) | 98.3 (2.0) | 98.5 (2.1) | 60.0 (NA) | 99.0 (NA) |
| TBI(+)-CVVH(+) | 98.8 (1.6) | 97.8 (4.3) | 98.5 (1.3) | 99.0 (0.7) | 98.6 (1.5) |
| KW (p) | 0.15 (0.93) | 1.15 (0.56) | 0.25 (0.88) | 2.4 (0.1) | 0 (1) |
|  | **ETCO_2_-1** | **ETCO_2_-2** | **ETCO_2_-3** | **ETCO_2_-4** | **ETCO_2_-5** |
| TBI(+)-CVVH(-) | 31.8 (7.1) | 26.1 (3.2) | 28.2 (4.4) | NA | NA |
| TBI(-)-CVVH(+) | 31.3 (8.0) | 30.0 (11.7) | 29.5 (12.0) | 32.0 (NA) | 33.0 (NA) |
| TBI(+)-CVVH(+) | 31.0 (5.4) | 28.5 (8.4) | 28.4 (10.1) | 28.6 (9.6) | 33.0 (7.8) |
| KW (p) | 0.04 (0.97) | 0.4 (0.81) | 0.09 (0.95) | 0.25 (0.6) | 0.1 (0.7) |

**Supplemental Table 2.** Hemofiltration time. TT = Total time of hemofiltration (min). ET = efficient time of hemofiltration (min). TBI(-)-CVVH(+) = Hemofiltration control group. TBI(+)-CVVH(+) = Traumatic brain injury group with hemofiltration. Values expressed as $\overline{\boldsymbol{x}}$(SD). MW = Mann-Whitney test.

| GROUP | TT | ET |
| --- | --- | --- |
| TBI(-)-CVVH(+) | 74.1 (29.5) | 57.5 (36.1) |
| TBI(+)-CVVH(+) | 105 (53) | 74.2 (27.9) |
| MW (p) | 22 (0.14) | 29.5 (0.36) |

**Supplemental Table 3.** Neurological Evaluation Score (NES) immediately (NES1) and 24 hr (NES2) after TBI for all groups. Values expressed as $\overline{\boldsymbol{x}}$±SD. TBI(+)-CVVH(-) = Traumatic brain injury group (n=9). TBI(-)-CVVH(+) = Hemofiltration group (n=6). TBI(+)-CVVH(+)= Combined traumatic brain injury group adding all rabbits with hemo-filtration before and after the trauma (n total=13). *p = 0.0020 between TBI(+)-CVVH(-) and CV (n= 6) †p = 0.02 between TBI(-)-CVVH(+)and TBI(+)-CVVH(+) using KW with Dunn test. KW= Kruskal-Wallis test

| GROUP | NES1 | NES2 |
| --- | --- | --- |
| TBI(+)-CVVH(-) | 2.6 (1.8) | 6.1 (1.4) |
| TBI(-)-CVVH(+) | 9.3 (0.81)* | 9.8 (0.40)* |
| TBI(+)-CVVH(+) | 4.0 (2.7)† | 7.5 (0.70) |
| KW (p) | 11.3 (0.003) | 10.3 (0.01) |

**Supplemental Table 4**. Proportional table (%) showing Pearson’s chi-squared between groups for the presence of cerebral ischemia ( p < 0.001), inflammation (p < 0.001), gliosis (p =0.14), haemorrhage (p < 0.001) and edema (p <0.001). *Denotes a significant standardized residual. TBI(+)-CVVH(-) = Traumatic brain injury group (n=5). TBI(-)-CVVH(+) = Hemofiltration group (n=2). TBI(+)-CVVH(+)= Combined traumatic brain injury group adding all rabbits with hemo-filtration before and after the trauma (n =9)

| PATHOLOGY | TBI(+)-CVVH(-) | TBI(-)-CVVH(+) | TBI(+)-CVVH(+) |
| --- | --- | --- | --- |
| Areas of ischemia |  |  |  |
| Mild | 16.6 | 0.0 | 83.3* |
| Severe | 40 | 20 | 40 |
| Areas of inflammation |  |  |  |
| No inflammation | 33.3 | 0.0* | 66.6 |
| Inflammation | 30.7 | 15.3 | 53.8 |
| Areas of gliosis |  |  |  |
| No gliosis | 37.5 | 12.5 | 50.0 |
| Gliosis | 25.0 | 12.5 | 62.5 |
| Areas of haemorrhage |  |  |  |
| No haemorrhage | 40.0 | 40.0 | 20.0 |
| Haemorrhage | 27.2 | 0.0 | 72.7* |
| Areas of edema |  |  |  |
| No edema | 80.0 | 50.0 | 33.3 |
| Edema | 20.0 | 50.0 | 66.6* |
